# Valence of Temporal Self-Appraisals: A Comparison Between First-Person Perspective and Third-Person Perspective

**DOI:** 10.3389/fpsyg.2021.778532

**Published:** 2021-11-11

**Authors:** Caizhen Yue, Yihong Long, Chaomei Ni, Chunhua Peng, Tong Yue

**Affiliations:** ^1^College of National Culture and Cognitive Science, Guizhou Minzu University, Guiyang, China; ^2^Laboratory of Emotion and Mental Health, Chongqing University of Arts and Sciences, Chongqing, China; ^3^Faculty of Psychology, Southwest University, Chongqing, China

**Keywords:** temporal self-appraisal, first-person perspective, third-person perspective, self-reference paradigm, self-positive bias

## Abstract

Mental time travel is one of the most remarkable achievements of mankind. On the one hand, people perceive past self, present self, and future self as a continuous unity; on the other hand, people have the ability to distinguish among the three types of temporal selves because there are different representations of them. In this study, we used an adapted temporal self-reference paradigm to explore the processing mechanism of different temporal selves. Temporal self-reference was performed from the first-person perspective in Experiment 1 and from the third-person perspective in Experiment 2. The results indicated that people showed a more positive bias toward future self compared with past self and present self no matter in the first-person perspective or third-person perspective. There was no difference in recognition rate among past self, present self, and future self. Compared with the first-person perspective, present self-processing in the third-person perspective was more abstract and generalized, which may reflect that the third-person perspective has the same distancing function as time. This study can deepen understandings on temporal self-appraisals from different perspectives.

## Introduction

One of the most remarkable achievements of mankind is able to perform mental time travel (MTT), which refers to people’s ability to mentally project oneself into the past or future (Endel, 2002; Rasmussen and Berntsen, 2013). MTT allows people to subjectively locate selves to the past time and places to reexperience their past, or to a future point-in-time to experience certain events (Liu et al., 2010). This shows that the self has temporal extension, possessing not only the present, but also the past and the future. Thus, MTT results in temporal self (Luo et al., 2013), which refers to past self, present self, and future self.

A person’s past experience will affect his or her self-knowledge, and individual past experience and knowledge on past self will be gradually internalized by the individual, becoming a part of self-identity. Similarly, the individual’s view of future self and imagination of future life will also affect the individual’s present behaviors and bring relevant information into the self-identity (D’Argembeau et al., 2012; Rubianes et al., 2021). Individuals perceive past self, present self, and future self as a unified and continuous unity, forming a stable self-identity (Northoff, 2017). For another, continuity does not mean the identical, and self-knowledge will be updated and reshaped as life circumstances constantly change. Therefore, in addition to feeling the temporal self-continuity, the ability to distinguish past self, present self, and future self is also an important part of self-processing (D’Argembeau et al., 2008).

Since human beings are capable of conducting MTT, individuals can project selves to different points in time, which means that there are different temporal distances correlated with the present, such as near distance (1month later) and far distance (5years later; Wilson et al., 2012; Luo et al., 2013). Studies have found that temporal distance is an important factor affecting people’s self-perception. According to construal level theory (CLT; Trope and Liberman, 2003; Eyal et al., 2008), near self is associated with a low level of concrete construal, while distant self is associated with a high level of abstract construal. In more detail, near self is based more on concrete events and contains complex and contextualized self-representations; while distant self is characterized by abstraction, schema thinking, and textualization. This means that present self should be different from past self and future self. Studies in this area also found that people often treat past self and future self as “others” compared with present self (Pronin, 2008; Yang et al., 2020). A study of the neural mechanisms of temporal self also found that cortical midline structures (CMSs) were activated when participants reflected on present self, compared with past self or future self (D’Argembeau et al., 2010). These studies showed that people’s present self-representations were more specific and contextual; while they were more abstract and generalized for distant past self and future self (Liberman et al., 2002; Wakslak et al., 2008).

As for the relevance between past self and future self, it has been found that the recall on past events can provide information for future self (Tanguay et al., 2020); many brain regions that support to memorize past events are also involved (Szpunar et al., 2007; Addis et al., 2009) when imagining future events that may happen. Studies in a wide range of fields have shown there was a striking similarity between remembering past events and imagining future events (D’Argembeau and Mathy, 2011; Szpunar et al., 2012), which suggested that the two temporal directions depended on a shared neural network and similar cognitive structure. Although past self and future self are very similar, there are important differences. Past events are something that happened, and the recall on them is limited by what happened; while future events do not happen and are more affected by imagining process (Perrin, 2016; Malek et al., 2017). That is, the recall on the past represents our ability to reexperience the past, while the future is the ability to “experience future in advance” by simulating it in our mind (Gilbert and Wilson, 2007). Thus, the mental structure of future events is cognitively more demanding than the corresponding structure of past events, for example, past events score higher on measures related to the recall (such as imagery and vividness), while future MTT is related to more schema-based constructions, future events score higher on variables related to self-schema and abstract knowledge (Berntsen and Jacobsen, 2008; Berntsen and Bohn, 2010; D’Argembeau and Mathy, 2011; Miles and Berntsen, 2011). Future MTT correlates with more brain activities than past MTT (Szpunar et al., 2007; Addis et al., 2009).

In addition to temporal distance, treating oneself from different perspectives can also affect how people perceive selves. Studies have found that the third-person perspective also has the distancing function compared with the first-person perspective (D’Argembeau and Van der Linden, 2004; Sutin and Robins, 2008), which leads people to interpret events at an abstract level (Libby et al., 2005). For example, compared with the first-person perspective, observing a person’s behaviors from the third-person perspective may promote people to evaluate their own behaviors more objectively (Zhou et al., 2013), obtain lower emotional experience (Berntsen and Rubin, 2006), and reduce egocentric bias (Zhou et al., 2013). Some existing studies have shown that distant events are considered to have a different self-concept from current events, which leads that they are more often represented from the third-person perspective, and contain less sensory and contextual details (Libby and Eibach, 2002); similarly, future self is usually imagined from the third-person perspective (Pronin and Ross, 2006). This means that nearer present self tends to be viewed from the first-person perspective, while more distant past self or future self is usually viewed from the third-person perspective (Broemer et al., 2008).

Different temporal selves are involved in self-processing no matter from the first-person perspective or third-person perspective. One of the most common and forceful findings in this area is the self-positive bias (Watson et al., 2007; Chen et al., 2014), which refers to people’s tendency to view oneself with an unrealistically positive attitude, that is, we generally think we have more positive (and less negative) traits and abilities (Fields et al., 2019). For example, studies have found that people generally recognize positive personality traits, reject negative personality traits, and rate positive trait adjectives as self-relevant, but rate negative trait adjectives as non-self-relevant, people tend to respond faster to self-positive adjectives than to negative adjectives (Watson et al., 2007; Chen et al., 2014). Moreover, the people’s neural mechanism of the valence processing of self-relevant trait adjectives is not completely the same (Fossati et al., 2003; Brühl et al., 2014; van der Cruijsen et al., 2018; Zhang et al., 2020). This implies that self-positive bias not only reflects positive self-concept, but also indicates that there are differences in the processing of valence of different self-relevant trait adjectives.

People judge oneself positively at different points-in-time, but the positive degree of self can vary with different points-in-time. Temporal self-appraisal theory believes that people’s appraisals on past self make them feel good about present self; in order to maintain positive self-view, people often tend to devalue past self. That is, even if there is no actual improvement in present self, people will make oneself feel better by devaluing past self. Temporal self-appraisal theory is supported by many researches. As opposed to devaluing past self, people usually view future self in a more positive light (Hershfield, 2011; Szpunar et al., 2012), which indicates that people think they are getting better and better over time (Yang et al., 2017). Positive bias toward future self is also supported by a number of studies, for example, positive future events are remembered in more detail than negative future events (Gallo et al., 2011), and future events are rated as more emotionally positive than past events (Berntsen and Bohn, 2010), even as depressed individuals, they are very optimistic about their future selves (Sokol and Serper, 2017). These studies showed that, relatively speaking, people are the least positive toward past self, put present self in the middle, and are the most positive toward future self.

Studies on self-reference showed that people encode self-relevant information more deeply, which is better than processing information about others (Fossati et al., 2004). Based on previous studies, people often treat past self and future self as “others” (Pronin, 2008), does that mean the individual more deeply processes present self compared with past self and future self? Given the positive bias of future self, will this positive bias be reflected not only in trait adjectives ratings, but also in the recognition task of trait adjectives? Because of the distancing mechanism of the third-person perspective, what is the difference on evaluating past self, present self, and future self between the third-person perspective and the first-person perspective? Based on this, this study adopted the revised temporal self-reference paradigm (specifically, participants were asked to conduct trait adjectives ratings on past self, present self, and future self, and received a surprising recognition task; Conway and Dewhurst, 1995; Yue et al., 2020), and used two experiments conducted from the first-person perspective and the third-person perspective to answer the above questions. We predicted that the recognition results of present self are better than those of past self and future self; whether in trait adjectives ratings or in the recognition, they show greater positive bias for future self. Due to the distancing mechanism in the third-person perspective, present self-processing in the third-person perspective may be different from that in the first-person perspective; specifically, we predicted that present self would be more positive than past self in the first-person perspective; while present self-processing was similar to past self and did not show any more positive bias than past self in the third-person perspective.

## Experiment 1

Experiment 1 was conducted to investigate the memory effect of temporal self-appraisals from the first-person perspective. Participants were asked to make trait adjectives judgment of past self (the self 5years ago), present self, and future self (the self 5years later) from the first-person perspective and conducted a surprising recognition task. We predicted the positive bias of future self in trait adjectives ratings and recognition rates; in addition, the recognition rate of present self was higher than that of past self and future self.

### Methods

#### Participants

In this within-participants-design experiment, we estimated the required sample size by using *f*=0.27 as effect size input in G-Power 3.1.9 (α=0.05; Faul et al., 2007) to detect a medium-sized effect on the main outcomes. The calculation outcome suggested a required sample size of 24 for each experiment in this study. In Experiment 1, we recruited 37 healthy undergraduates (10 males and 27 females, with a mean age of 21.19years; *SD*=0.97) to participate in our study. All participants had normal or corrected-to-normal visions. They did not have a history of neurological disorders. All participants provided written informed consent, in accordance with the Declaration of Helsinki.

#### Materials

We selected a set of 240 Chinese adjectives to compose a list of stimuli for the encoding and recognition phases. The pleasure, meaningfulness, familiarity, and valence of the adjectives were considered and balanced based on the norms of Wang (2005). In all, 120 adjectives were presented at the encoding phase and the other 120 adjectives were used as lures at recognition. The study words were divided into six sub-lists (40 words each, 20 positive and 20 negative), matched on the basis of familiarity, meaningfulness, and pleasure from the norms of Wang (2005). These materials have been used in previous studies (Yue et al., 2020), there were no significant differences on pleasure, meaningfulness, and familiarity across the six groups of adjectives, there were significant differences in the valence (positive vs. negative) in each sub-list. The number of characters in each sub-list of adjectives is equal (each adjective is composed of two to four Chinese characters). Each sub-list was assigned to one of three encoding conditions (past self condition, present self condition, or future self condition) and counterbalanced across participants. Each sub-list was composed of half positive (e.g., generous and pleasant) and half negative (e.g., jealous and rude) traits. The order of presentation was randomized for each adjective for each participant, with trials from different conditions intermixed throughout the study.

The Inclusion of Other in the Self (IOS) scale (Aron et al., 1991) was used to measure the closeness of temporal self. The IOS scale consists of seven pairs of overlapping circles, with each pair overlapping slightly more than the preceding pair. The participants were asked to select the pair of circles that best portrays the relationship between past self (future self) and present self. Meanwhile, the seven-point scale was adopted to evaluate the frequency of recalling the past and imagining the future recollections (1=never recall or imagine, 7=very frequent).

#### Measures

To understand the positive self-view of participants, we took the practices (Sokol and Serper, 2017) to use the mean value of all positive adjective ratings (response times, recognition rates) of each participant plus the mean value of negative adjectives multiplied by (−1), to create a completion measure of a “positive self-view” that combines negative and positive adjectives. This mathematical process created three independent variables for the past, present, and future, respectively, namely past self-view, present self-view, and future self-view. Because negative adjectives were scored in reverse and combined with positive adjectives, variable scores can be positive or negative value.

#### Procedures

In the questionnaire stage, after the participants sat down in the laboratory, they were first asked to fill in the IOS scale. They rated the relation degree of the self 5years ago and present self, as well as of present self and the self 5years later. According to Ersner-Hershfield et al. (2009) and Liu et al. (2018), the IOS scale after adapting could effectively measure temporal self-continuity; participants were then asked to rate the frequency of recalling the past and imagining the future, after filling out the questionnaires, they were asked to perform in the experiment stage.

##### Encoding Task and Recognition Task

Participants practiced the encoding task to ensure that they understood the demands of the task. Then, they received instructions to encode adjectives in one of the three ways: past self condition (e.g., was I kind 5years ago?), present self condition (e.g., am I kind now?), or future self condition (e.g., will I be kind 5years later?). Every way represented one of the three conditions. Following a 4-s presentation of the adjectives, participants were asked to press the D key for “Always like this,” F key for “Generally like this,” J key for “Generally not like this,” and K key for “Never like this.” 120 adjectives were encoded in the task phase, and 40 adjectives (20 positive and 20 negative) were assigned to each of the three conditions. The presentation orders of different trials and conditions were counterbalanced across participants. After encoding task, participants answered a Raven’s Standard Progressive Matrices test, starting from the “C” section for 6min, to eliminate the effect of memory of the encoding task. They then decided if the adjective presented on the center of the screen had been shown in the previous task, by pressing the F key to classify it as an old word or pressing the J key to classify it as a new word. In this task, a total of 240 adjectives were tested: 120 adjectives were tested as encoded words that had been presented in the previous task, and another 120 adjectives were tested as new words. All stimuli presentation and behavioral response collection were controlled by E-prime 2.0, running on a 17-inch DELL LED display with a resolution of 1,024×768 and a refresh rate of 60Hz.

### Results

#### IOS Ratings and Frequency Ratings

The paired samples t-test indicated that there was no significant difference in IOS Ratings between past–present (*M*=4.22, *SD*=1.16) and present–future (*M*=4.27, *SD*=1.43), *t*(36)=0.25, *p*=0.81. A paired samples t-test was conducted using frequency as the dependent variable. Future frequency imagined by participants (*M*=5.32, *SD*=0.92) was significantly higher than frequency of recalling the past (*M*=4.76, *SD*=1.30), *t*(36)=2.57, *p*<0.05, *r*=0.24.

#### Four-Point Ratings in the Encoding Task

The 4-point rating scores were analyzed by a 3×2 repeated-measures ANOVA, with the encoding condition (past self vs. present self vs. future self) and valence of the adjectives (negative vs. positive). The main effect of the encoding condition was significant [*F*(2,72)=4.24, *p*<0.05, $\eta_{p}^{2}\;$=0.11]. The main effect of the valence of the adjectives was also significant [*F*(1, 36)=115.56, *p*<0.001, $\eta_{p}^{2}$=0.76]. The interaction effect of encoding condition×valence of the adjectives was significant [*F*(2,72)=13.76, *p*<0.001, $\eta_{p}^{2}$=0.28]. We performed simple effects analyses when this interaction was observed. The results showed that the positive valence scores were significantly higher than the negative valence scores of adjectives in the past self-condition [*F*(1,36)=43.56, *p*<0.001, $\eta_{p}^{2}$=0.55], present self-condition [*F*(1,36)=95.64, *p*<0.001, $\eta_{p}^{2}$=0.73], and future self-condition [*F*(1, 36)=110.59, *p*<0.001, $\eta_{p}^{2}$=0.75] (see Table 1).

**Table 1:** Descriptive statistical results of trait ratings, response times (ms) of trait ratings, and recognition under the first-person perspective.

		Past self	Present self	Future self
Trait ratings	Positive	3.01 (0.37)	3.03 (0.34)	3.14 (0.42)
	Negative	2.35 (0.41)	2.21 (0.32)	2.00 (0.36)
Response times	Positive	1,724 (478)	1,698 (485)	1,489 (418)
	Negative	1,847 (470)	1,768 (491)	1,763 (480)
Recognition	Positive	0.77 (0.16)	0.78 (0.17)	0.81 (0.14)
	Negative	0.77 (0.17)	0.75 (0.17)	0.72 (0.16)

**Figure 1:**
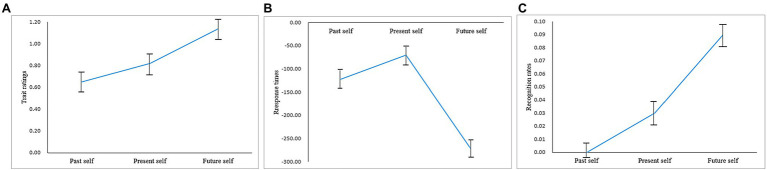
Positive bias of different temporal selves under the first-person perspective, **(A)** trait ratings; **(B)** response times (ms) of trait ratings; **(C)** corrected recognition rates.

*The one-way repeated-measures ANOVA showed that the rating scores of positive self-views were significantly different [F(2,72)=13.76, *p*<0.001, $\eta_{p}^{2}$=0.28]. The *post hoc* analysis showed that future self scores’ difference (M=1.14, SD=0.66) were significantly higher than present self (M=0.82, SD=0.51) and past self (M=0.65, SD=0.60; ps<0.05), and present self was higher than past self (p=0.06; see Figure 1A)*.

#### Response Times in the Encoding Task

The response times were analyzed by a 3×2 repeated-measures ANOVA, with the encoding condition (past self vs. present self vs. future self) and valence of the adjectives (negative vs. positive). The main effect of the encoding condition was significant [*F*(2,72)=6.60, *p*<0.01, $\eta_{p}^{2}$=0.16]. The main effect of the valence of the adjectives was also significant [*F*(1,36)=17.38, *p*<0.001, $\eta_{p}^{2}$=0.33]. The interaction effect of encoding condition×valence of the adjectives was significant [*F*(2,72)=9.17, *p*<0.001, $\eta_{p}^{2}$=0.20]. We performed simple effects analyses when this interaction was observed. The results showed that the positive valence RTs were significantly higher than the negative valence RTs of adjectives in the past self condition [*F*(1,36)=6.49, *p*<0.05, $\eta_{p}^{2}$=0.15] and future self condition [*F*(1,36)=33.51, *p*<0.001, $\eta_{p}^{2}$=0.48]. There was no significant difference between positive and negative valence RTs in the present self-condition [*F*(1,36)=2.39, *p*=0.13].

The one-way repeated-measures ANOVA showed that the RTs of positive self-view were significantly different [*F*(2,27)=9.17, *p*<0.001, $\eta_{p}^{2}$=0.20]. The *post hoc* analysis showed that the past self RTs difference (*M*=−70.05, *SD*=275.70) was significantly higher than the present self (*M*=−122.57, *SD*=292.77) and future self (*M*=−273.60, *SD*=287.51; *ps*<0.05). There was no significant difference between past self and present self (*p*=0.26; see Figure 1B).

#### Recognition Memory Performance

The corrected recognition scores were analyzed by a 3×2 repeated-measures ANOVA, with the encoding condition (past self vs. present self vs. future self) and valence of the adjectives (negative vs. positive). The main effect of the encoding condition was not significant [*F*(2,72)=0.24, *p*=0.79]. The main effect of the valence of the adjectives was significant [*F*(1,36)=4.43, *p*<0.05, $\eta_{p}^{2}$=0.11]. The interaction effect of encoding condition×valence of the adjectives was significant [*F*(2,72)=4.20, *p*<0.05, $\eta_{p}^{2}$=0.11]. We performed simple effects analyses when this interaction was observed. The results showed that the positive valence recognition scores were significantly higher than the negative valence recognition scores of adjectives in the future self condition [*F*(1,36)=16.20, *p*<0.001, $\eta_{p}^{2}$=0.31]. There was no significant difference between positive and negative valence recognition scores in the past self condition [*F*(1,36)=0.00, *p*=0.96] and present self-condition [*F*(1,36)=0.94, *p*=0.34].

The one-way repeated-measures ANOVA showed that the corrected recognition scores of positive self-views were significantly different [*F*(2,27)=4.20, *p*<0.05, $\eta_{p}^{2}$=0.105]. The *post hoc* analysis showed that the future self recognition scores difference (*M*=0.09, *SD*=0.14) was significantly higher than the past self (*M*=0.00, *SD*=0.16) and present self (*M*=0.03, *SD*=0.18; *ps*<0.05). There was no significant difference between past self and present self (*p*=0.42; see Figure 1C).

### Discussion

The results of experiment 1 showed that, in terms of explicit closeness, the participants thought the closeness between past self and present self was the same as the closeness between present self and future self. But people imagined the future more frequently than they recalled the past. This study also found that there was no difference in recognition rate among the three temporal selves, that is, the recognition rates of past self, present self and future self were the same, which did not verify our hypothesis.

As for the positive bias of self, this study found that in trait adjectives ratings, the score of positive self-appraisals was significantly higher than that of negative self-appraisals. The response times of positive trait adjectives in past self and future self were faster than that of negative trait adjectives, and there was no difference between positive trait adjectives and negative trait adjectives in present self condition. This result may reflect the effect of temporal distance. In terms of recognition rates, positive trait adjectives in the future self condition were significantly higher than negative trait adjectives, while there was no difference in recognition rate between positive and negative trait adjectives in the past self-condition and present self-condition, which may indicate that future self-concept is more composed of positive traits than past self and present self. Experiment 1 also compared the degree of positive self under three temporal self conditions. The positive bias of future self in trait adjectives rating scores, response times of trait adjectives ratings and recognition rate were larger, indicating that people were more optimistic and positive about future self.

## Experiment 2

The results of experiment 1 found that future self preformed positive bias in the first-person perspective, and the recognition scores of past self, present self and future self were basically equal, and there were no significant differences among them. The third-person perspective was used in experiment 2 to verify the results of Experiment 1. It seems to be a kind of distancing mechanism due to the function of the third-person perspective (D’Argembeau and Van der Linden, 2004; Sutin and Robins, 2008). Existing researches showed that the distant events usually adopt the third-person perspective, which meant when viewing long-distance past self (the self 5years ago) and future self (the self 5years later), there was little difference by adopting the first-person and the third-person perspectives, while there was difference by adopting the first-person and the third-person perspectives by viewing present self.

### Methods

#### Participants

In Experiment 2, we recruited 37 healthy undergraduates (16 males and 21 females, with a mean age of 20.95years; *SD*=1.03) to participate in our study. All participants had normal or corrected-to-normal visions. They did not have a history of neurological disorders. All participants provided written informed consent, in accordance with the Declaration of Helsinki.

#### Materials, Measurements, and Procedures

The materials and procedures were identical to Study 1. Only three experimental conditions were used in the third-person perspective, specifically, past self (e.g., do people think I was kind before 5years?), present self (e.g., do people think I am kind now?), and future self (e.g., do people think I’ll be kind in 5years?).

### Results

#### IOS Ratings and Frequency Ratings

The paired samples t-test indicated that there was no significant difference in IOS Rating between past–present (*M*=4.49, *SD*=1.35) and present–future (*M*=4.32, *SD*=1.23), *t*(36)=0.52, *p*=0.61. A paired samples t-test was conducted using frequency as the dependent variable. Future frequency imagined by participants (*M*=5.27, *SD*=1.09) was significantly higher than frequency of recalling the past (*M*=4.81, *SD*=1.39), *t*(36)=2.26, *p*<0.05, *r*=0.18.

#### Four-Point Ratings in the Encoding Task

The four-point rating scores were analyzed by a 3×2 repeated-measures ANOVA, with the encoding condition (past self vs. present self vs. future self) and valence of the adjectives (negative vs. positive). The main effect of the encoding condition was not significant [*F*(2,72)=0.72, *p*=0.49]. The main effect of the valence of the adjectives was significant [*F*(1,36)=144.19, *p*<0.001, $\eta_{p}^{2}$=0.80]. The interaction effect of encoding condition×valence of the adjectives was significant [*F*(2,72)=8.774, *p*<0.001, $\eta_{p}^{2}$=0.20]. We performed simple effect analyses when this interaction was observed. The results showed that the positive valence scores were significantly higher than the negative valence scores of adjectives in the past self-condition [*F*(1,36)=74.09, *p*<0.001, $\eta_{p}^{2}$=0.67], present self-condition [*F*(1,36)=79.41, *p*<0.001, $\eta_{p}^{2}$=0.69], and future self-condition [*F*(1,36)=127.35, *p*<0.001, $\eta_{p}^{2}$=0.78] (see Table 2).

**Figure 2:**
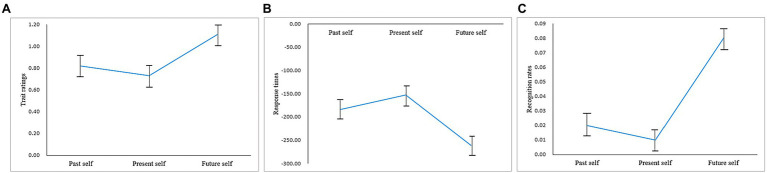
Positive bias of different temporal selves under the third-person perspective, **(A)** trait ratings; **(B)** response times (ms) of trait ratings; **(C)** corrected recognition rates.

The one-way repeated-measures ANOVA showed that the rating scores of positive self-view were significantly different [*F*(2,72)=8.77, *p*<0.001, $\eta_{p}^{2}$=0.20]. The *post hoc* analysis showed that the future self scores difference (*M*=1.11, *SD*=0.60) was significantly higher than the past self (*M*=0.82, *SD*=0.58) and present self (*M*=0.73, *SD*=0.50; *ps*<0.05). There was no significant difference between the past self and present self (*p*>0.05; see Figure 2A).

**Table 2:** Descriptive statistical results of trait ratings, response times (ms) of trait ratings, and recognition under the third-person perspective.

		Past self	Present self	Future self
Trait ratings	Positive	3.00 (0.38)	2.93 (0.32)	3.12 (0.35)
	Negative	2.19 (0.29)	2.20 (0.31)	2.01 (0.32)
Response times	Positive	1,707 (433)	1,752 (433)	1,620 (475)
	Negative	1,892 (460)	1,904 (463)	1,881 (505)
Recognition	Positive	0.81 (0.16)	0.81 (0.12)	0.85 (0.11)
	Negative	0.79 (0.13)	0.80 (0.13)	0.77 (0.17)

#### Response Times in the Encoding Task

The response times were analyzed by a 3×2 repeated-measures ANOVA, with the encoding condition (past self vs. present self vs. future self) and valence of the adjectives (negative vs. positive). The main effect of the encoding condition was not significant [*F*(2,72)=0.91, *p*=0.41]. The main effect of the valence of the adjectives was significant [*F*(1,36)=49.57, *p*<0.001, $\eta_{p}^{2}$=0.58]. The positive valence RTs were significantly higher than the negative valence RTs of adjectives in the three-encoding condition. The interaction effect of encoding condition×valence of the adjectives was not significant [*F*(2,72)=1.98, *p*=0.15]. We performed simple effects analyses when this interaction was observed. The results showed that the positive valence RTs were significantly higher than the negative valence RTs of adjectives in the past self-condition [*F*(1,36)=19.89, *p*<0.001, $\eta_{p}^{2}$=0.36], present self-condition [*F*(1,36)=18.01, *p*<0.001, $\eta_{p}^{2}$=0.33], and future self-condition [*F*(1,36)=26.64, *p*<0.001, $\eta_{p}^{2}$=0.43].

The one-way repeated-measures ANOVA showed that the RTs of positive self-view were not significantly different [*F*(2,72)=1.98, *p*=0.15] (see Figure 2B).

#### Recognition Memory Performance

The corrected recognition scores were analyzed by a 3×2 repeated-measures ANOVA, with the encoding condition (past self vs. present self vs. future self) and valence of the adjectives (negative vs. positive). The main effect of the encoding condition was not significant [*F*(2,72)=0.12, *p*=0.88]. The main effect of the valence of the adjectives was significant [*F*(1,36)=6.08, *p*<0.05, $\eta_{p}^{2}$=0.15]. The interaction effect of encoding condition×valence of the adjectives was significant [*F*(2,72)=3.39, *p*<0.05, $\eta_{p}^{2}$=0.09]. We performed simple effect analyses when this interaction was observed. The results showed that the positive valence recognition scores were significantly higher than the negative valence recognition scores of adjectives in the future self condition [*F*(1,36)=11.65, *p*<0.01, $\eta_{p}^{2}$=0.24]. There was no significant difference between positive and negative valence recognition scores in the past self condition [*F*(1,36)=0.59, *p*=0.45] and present self condition [*F*(1,36)=0.32, *p*=0.58].

The one-way repeated-measures ANOVA showed that the corrected recognition scores of positive self-views were significantly different [*F*(2,72)=3.39, *p*<0.05, $\eta_{p}^{2}$=0.09]. The *post hoc* analysis showed that the future self recognition scores difference (*M*=0.08, *SD*=0.14) was significantly higher than past self (*M*=0.02, *SD*=0.13) and present self (*M*=0.01, *SD*=0.13; *ps*<0.05). There was no significant difference between past self and present self (*p*=0.89; see Figure 2C).

### Discussion

The results of Experiment 2 basically verified the results of experiment 1. Specifically, people imagined the future more frequently, and there was no difference in the recognition rate of the three temporal selves, and they showed positive bias toward future self. But experiment 2 also found some interesting differences from experiment 1, mainly in present self. This may reflect the distancing function of the third-person perspective.

## General Discussion

This study found that, compared with past self and present self, people showed a greater positive bias toward future self, which was reflected not only in the response times of trait adjectives ratings, but also in the recognition rates. Contrary to our hypothesis, our study found there was no difference in recognition rates of past self, present self, and future self in the temporal self-reference paradigm. Present self-processing was similar to distant self-processing under the third-person perspective (D’Argembeau and Van der Linden, 2004) and did not show more positive bias than past self, which was different from the first-person perspective.

On explicit measures of closeness, the results showed no difference between past–present closeness and present–future closeness. But on the frequency, people imagined the future more frequently than they recalled the past. This result was consistent with the findings of previous studies (Smallwood et al., 2009). Studies have found that people thought about the future much more than they did in the past (Anderson and McDaniel, 2019), and the frequency of thinking about the future was about three and a half times as high as they did about the past (Baumeister et al., 2020). Researchers suggested that future thinking, especially goal processing (Ernst et al., 2018), may be a core component of human thinking (Suddendorf et al., 1997), which should reflect that thinking about the future is more adaptive than thinking about the past (Baumeister et al., 2020), and thinking about the future will prompt individuals to prepare for future actions, including decisions and executive needs (Baumeister et al., 2016).

Contrary to our predictions, the two experiments in this study found there was no difference in recognition rates between past self, present self, and future self. Studies on the self-reference effect have found that although we have an advantage in processing self-relevant information (Symons and Johnson, 1997), our cognitive processing of close others is as good as that of selves (Mashek et al., 2003; Lee et al., 2016), which indicated that people can incorporate the views and information of close others into the self (Aron et al., 1991; Ketay et al., 2019). The results of this study suggested that even when we see the distance between the past and the future as the “others” (Pronin, 2008), similarly, we see the distant self as the “close others.” In fact, our selves involve temporal extension from the present to the past and the future (Northoff, 2017).

The results of this study also expanded the research on self-positive bias (Fields et al., 2019). No matter in past self, present self, or future self, people all tended to rate positive traits as self-relevant, while negative traits as self-irrelevant. The response times to positive trait adjectives were faster than those to negative trait adjectives. Consistent with previous research results (Sharot et al., 2007), this study also found that although positive self-bias existed in both the first-person and the third-person perspectives, positive self-bias will be larger in the future. In other words, people were more positive and optimistic about their future selves (Newby-Clark and Ross, 2003), which reflected that people liked to perceive their continuous progresses. Different from past self and present self, people’s recognition rates of future self were higher than those of negative self, indicating that people’s future self-concept was composed of more positive traits. This suggested that people’s positive bias toward future self was not only reflected in emotions, but also in the cognitive structure of future self, which may indicate that we were free to perceive future self based on our wishes, hopes, and plans (Pronin and Ross, 2006), and this positive memory would help build personal and social resources (Talarico et al., 2008).

Another interesting finding of this study was that present self-processing in the first-person perspective was different from present self-processing in the third-person perspective. Specifically, from the first-person perspective, there was no difference between the response times to positive trait adjectives and negative trait adjectives of present self, while the response times to the positive trait adjectives were faster than the negative trait adjectives of past self and future self. In the third-person perspective, positive trait adjectives of past self, present self, and future self were judged faster than negative trait adjectives. In addition, present self in the first-person perspective was higher than past self in the trait adjectives rating of positive self-view, indicating that present self was more positive than past self in the first-person perspective. In the third-person perspective, there was no difference between present self and past self, indicating that present self did not show more positive bias than past self. We thought this result reflects the distancing function of temporal distance and the third-person perspective. Present self-representation was more specific and contextualized from the first-person perspective (Wakslak et al., 2008), with more internal states and emotions (Broemer et al., 2008), and more realistic and variable (Wilson et al., 2012). Therefore, the cognitive processing of trait adjectives under present self condition was more complex. However, based on construal level theory (CLT; Trope and Liberman, 2003; Eyal et al., 2008), people’s representations of more distant past self and future self were more abstract and generalized and showed an ignorance for the internal state of the self (Pronin, 2008), activating a more general self-concept. Because positive information was more effective than negative information in attracting attention (Zhou et al., 2013), the response times of positive trait adjectives were faster than those of negative trait adjectives. We believed that this inference also applied to present self in the third-person perspective, because the third-person perspective had the same distancing function (D’Argembeau and Van der Linden, 2004; Sutin and Robins, 2008).

This study explored the positive bias of future self from the first-person and the third-person perspectives and found that the present self-processing in the third-person perspective was different from the present self-processing in the first-person perspective, which promoted our understanding of temporal selves. However, this study also has some limitations. First, in the selection of participants, previous studies have shown that the third-person perspective played a greater role in the self-development of adolescents (Crone and Fuligni, 2020; Yue et al., 2021). Therefore, whether the conclusions of this study can be extended to other participant groups needs to be further discussed. Second, in terms of perceiving contents, previous studies have found that, with the growth of individuals, people form more differentiated self-concept (academic, physical and prosocial, etc.), which are different in different fields and different social backgrounds (Harter, 2015; van der Cruijsen et al., 2018). Therefore, whether the conclusions of this study can be extended to different domains of self needs to be further studied. Third, previous studies have shown that the self can be divided into individual self, relational self, and collective self in terms of the selection of the perspectives of self (Sedikides et al., 2011), so whether the research conclusion of temporal self based on individual self can be applied to temporal self based on collective self also needs in-depth explorations (Topcu and Hirst, 2020).

In conclusion, this study found that people will imagine the future more frequently. In both the first-person and the third-person perspectives, people showed more positive bias toward future self than past self and present self. In recognition, there was no difference between past self, present self and future self, indicating that even if people treated past self and future self as “others,” we still treated past self and future self as “close others.” Present self of the third-person perspective was different from that of the first-person perspective due to the distancing function of the third-person perspective. The former was more abstract and generalized, while the latter was more specific and situational.

## Data Availability Statement

The raw data supporting the conclusions of this article will be made available by the authors, without undue reservation.

## Ethics Statement

The studies involving human participants were reviewed and approved by the Ethics Committee of Guizhou Minzu University agreed to carry out the study, and all the procedures involved were in line with the sixth revision of the Helsinki Declaration. The patients/participants provided their written informed consent to participate in this study.

## Author Contributions

CY and TY designed the experiments. CY, CN, and YL carried out the experiments. CY, CN, and CP analyzed the sequencing data. CY and YL drafted the initial manuscript and revised the manuscript. All authors approved the final manuscript as submitted and agreed to be accountable for all aspects of the work.

## Funding

This research was supported by the National Education Science Planning Project, China Western Project, “The mental mechanism of group identification for the adolescents in minority area,” Grant No. XMA200282.

## Conflict of Interest

The authors declare that the research was conducted in the absence of any commercial or financial relationships that could be construed as a potential conflict of interest.

## Publisher’s Note

All claims expressed in this article are solely those of the authors and do not necessarily represent those of their affiliated organizations, or those of the publisher, the editors and the reviewers. Any product that may be evaluated in this article, or claim that may be made by its manufacturer, is not guaranteed or endorsed by the publisher.
